# Value of three-dimensional visualization of preoperative prostatic magnetic resonance imaging based on measurements of anatomical structures in predicting positive surgical margin after radical prostatectomy

**DOI:** 10.3389/fendo.2023.1228892

**Published:** 2023-10-04

**Authors:** Bo Fan, Luxin Zhang, Yuchao Wang, Zhihong Dai, Heming Pan, Jiaxin Xie, Hao Wang, Zihan Xin, Yutong Wang, Xu Duan, Jiawen Luo, Liang Wang, Zhiyu Liu

**Affiliations:** ^1^ Department of Urology, Second Affiliated Hospital of Dalian Medical University, Dalian, Liaoning, China; ^2^ Liaoning Provincial Key Laboratory of Urological Digital Precision Diagnosis and Treatment, the Liaoning Provincial Department of Science and Technology, Dalian, Liaoning, China; ^3^ Liaoning Engineering Research Center of Integrated Precision Diagnosis and Treatment Technology for Urological Cancer, Liaoning Provincial Development and Reform Commission, Dalian, Liaoning, China; ^4^ Dalian Key Laboratory of Prostate Cancer Research, Dalian Science and Technology Bureau, Dalian, Liaoning, China; ^5^ Department of Scientific Research, Dalian Neusoft University of Information, Dalian, Liaoning, China; ^6^ Institute of Urology, Peking University, Beijing, China; ^7^ Department of Clinical Medicine, First Clinical School of Dalian Medical University, Dalian, Liaoning, China; ^8^ Department of Radiology, Second Affiliated Hospital of Dalian Medical University, Dalian, Liaoning, China

**Keywords:** prostate cancer, radical prostatectomy, positive surgical margin, magnetic resonance imaging, three-dimensional visualization

## Abstract

**Background:**

Positive surgical margin (PSM) or apical positive surgical margin (APSM) is an established predictive factor of biochemical recurrence or disease progression in prostate cancer (PCa) patients after radical prostatectomy. Since there are limited usable magnetic resonance imaging (MRI)-based models, we sought to explore the role of three-dimensional (3D) visualization for preoperative MRI in the prediction of PSM or APSM.

**Methods:**

From December 2016 to April 2022, 149 consecutive PCa patients who underwent radical prostatectomy were retrospectively selected from the Second Affiliated Hospital of Dalian Medical University. According to the presence of PSM or APSM, patients were divided into a PSM group (n=41) and a without PSM group (n=108) and into an APSM group (n=33) and a without APSM group (n=116). Twenty-one parameters, including prostate apical shape, PCa distance to the membranous urethra, and pubic angle, were measured on 3D visualization of MRI. The development of the nomogram models was built by the findings of multivariate logistic regression analysis for significant factors.

**Results:**

To predict the probability of PSM, a longer PCa distance to the membranous urethra (OR=0.136, p=0.019) and the distance from the anterior peritoneum to the anterior border of the coccyx (work space AP, OR=0.240, p=0.030) were independent protective factors, while a type 3 prostate apical shape (OR=8.262, p=0.025) and larger pubic angle 2 (OR=5.303, p=0.029) were identified as independent risk factors. The nomogram model presented an area under the curve (AUC) of the receiver operating characteristic curve (ROC) of PSM of 0.777. In evaluating the incidence of APSM, we found that the distance to the membranous urethra (OR=0.135, p=0.014) was associated with a low risk of APSM, while larger pubic angle 1 (OR=4.666, p=0.043) was connected to a higher risk of APSM. The nomogram model showed that the AUC of APSM was 0.755.

**Conclusion:**

As 3D visualization for preoperative MRI showed good performance in predicting PSM or APSM, the tool might be potentially valuable, which also needs to be validated by multicenter, large-scale, prospective studies.

## 1 Introduction

According to the Global Cancer Statistics 2020 record, there was predicted to be 1.4 million new cases of and 375,000 deaths from prostate cancer (PCa) worldwide in 2020. PCa ranks second in the incidence of male malignant tumors and fifth in mortality (1, 2). In recent years, with the acceleration of demographic aging, changes in dietary structure and improvements in PCa diagnostic technology, the incidence of PCa in China has shown a significant upward trend. The morbidity and mortality of PCa rank second and fifth for Chinese male malignant tumor incidence and mortality, respectively, and it is gradually becoming an important disease affecting the health of elderly men in China (1). There are various treatment options for PCa, including watchful waiting and active surveillance, radical prostatectomy (RP), and radical radiotherapy. RP is the preferred therapy for clinically confined PCa, including complete removal of the prostate and seminal vesicles. However, approximately 11%-40% of patients have been reported to have positive surgical margins (PSM) after undergoing RP, which is the highest PSM rate among male malignant tumors (3–5).

PSM is defined as the presence of tumor cells in postoperative pathological specimens that are in contact with pigment markers on the specimen surface. There are actually two types of PSM in clinical practice: one is when the cancer tissue has infiltrated outside the capsule, the tumor cannot be completely removed by surgery, and some cancerous tissue is left outside the prostate on the ink-stained margins; the other is when the cancer tissue is confined to the capsule, the fascia or capsule around the prostate is incised for various reasons, and enters the prostate gland by mistake, resulting in the disappearance of part of the fascia and capsule in the specimen, and the prostate remains on the ink-stained incision edge (6). The apex is the most frequent site of PSM due to the broad range of the dorsal venous complex, its location under the pubic bone approaching the neurovascular bundles, and the lack of capsule coverage. Surgeons preserve the urethral sphincter as much as possible to protect postoperative urinary control function and for many other reasons (7). PSM is considered to be an important factor leading to an elevated risk of biochemical recurrence and recurrence of local tumors, which occasionally require secondary treatment (4, 6). During 7-13 years of follow-up, 27-44% of patients diagnosed with PSM developed biochemical recurrence, 6.8-24.3% exhibited systemic disease progression, and 0.8-3.7% experienced cancer-related death (8–11). A meta-analysis of 32 studies involving 140,000 PCa cases showed that the overall survival rate of patients determined to have negative margins was 1.11 times that of patients determined to have PSM (12). Moreover, patients with PSM may experience severe depression due to excessive psychological stress (13). Although laparoscopic radical prostatectomy (LRP) and robot-assisted laparoscopic radical prostatectomy (RALRP) improve functional recovery after surgery compared to open radical prostatectomy, there is no remarkable efficacy in terms of PSM reduction. The occurrence of PSM after RP has become a common and difficult-to-ignore problem. Correctly diagnosing and evaluating PSM, reducing the incidence of PSM, and improving the therapeutic effect of radical prostatectomy are challenges that urgently need to be solved.

Significant patient-specific predictors such as age, body mass index, and prostate-specific antigen (PSA) have been identified associated with PSM (14). Treatment-specific factors correlating with the PSM have also been confirmed, proficiency in anatomical hierarchy and the experience of surgeons may be major determinants of the PSM (15). Preoperative neoadjuvant therapy also contribute to reduce the incidence of PSM, nevertheless the effectiveness is still debatable (16, 17). Tumor-specific characteristics including prostate volume, T stage and Gleason score exposed significant notable association with PSM (18, 19). As the evolving progress of reliable prognostic biomarkers in urological cancer, increasing number of biomarkers for predicting PSM have been discovered (2): immunohistochemical staining for Ki-67 may indicate high risk of biochemical recurrence in patients with PSM after radical prostatectomy (20). Meanwhile, moderate or strong heterogeneous nuclear ribonucleoprotein A1 (hnRNPA1) immunostaining was relevant to positive surgical margin and adverse tumor features (21). Nevertheless, low or negative expression of carcinoembryonic antigen-related cell adhesion molecule 1 (CEACAM1) have linked to PSM in patients with ERG fusion-positive PCa (22). In addition to the abnormal expression of genes or proteins which included BRCA1-associated protein-1 (BAP1), RNA-binding motif protein 3 (RBP) and Fas-associated death domain-containing protein phosphorylation (FADD) by immunohistochemical analysis, changes of mutational level are also strongly associated with PSM by fluorescence *in situ* hybridization analysis (2, 23–25). Kluth M et al. reported that Chromosome 16q deletion was linked to PSM whether ERG fusion in PCa patients was positive or negative (26).

Multiparameter MRI (mpMRI) has become the preferred method for the noninvasive diagnosis of PCa due to its high resolution for soft tissue and multifaceted and multiparametric imaging. MRI has played an important guiding role in clinical staging, postoperative efficacy evaluation and recurrence (27–30). Although the measurement of various parameters related to the prostate has been achieved in two-dimensional (2D) planes based on mpMRI, the recognition of the three-dimensional (3D) structure of the prostate and surrounding tissue and the spatial distance measurements are still far from satisfactory. To achieve a better understanding of anatomy, the 3D printing technique rose to the occasion, while the prohibitive cost has limited large-scale application so far. With the in-depth promotion of precision medicine and digital medicine, clinical medicine has entered the digital age. One of the important technologies in digital medicine is to use human medical image datasets to achieve 3D visualization through software. Compared with 2D images, which provide less complete information on the complexity of the pelvic anatomy, 3D visualization technique may enable better multiangle observation of the anatomical structure and the variations in blood vessels and surrounding tissues. Therefore, we aimed to determine the value of 3D visualization of preoperative prostatic MRI based on measurements of anatomical structures in predicting PSM and APSM after RP.

## 2 Materials and methods

### 2.1 Study population

A case−control study was conducted in the Second Affiliated Hospital of Dalian Medical University. Included were 149 patients consecutively confirmed by postoperative pathology as having PCa with preoperative complete mpMRI after excluding patients with a history of neoadjuvant therapy. They were enrolled from December 2016 to April 2022. According to the marginal status, the 149 patients were divided into two groups: patients with PSM and patients without PSM. According to the sites of PSM, the 149 patients were divided into the patients with APSM and patients without APSM two groups based on the presence or absence of APSM. All procedures in this study followed the Declaration of Helsinki and received the approval of the Ethics Review Committee of the Second Affiliated Hospital of Dalian Medical University. We explained to the enrolled patients that this study would not involve follow-up or harm their health and would keep their information confidential, and informed consent was obtained from all enrolled patients (ethical approval number: 202285).

### 2.2 Pathology

As recommended by the International Society of Urological Pathology (ISUP) Consensus Conference on Handling and Staging of Radical Prostatectomy Specimens, routine pathological examinations of RP specimens were performed (31). All cases were evaluated by professional pathologists. The extent of the tumor, stage and PSM locations were determined complying with the TNM system and PCa reporting and staging guidelines (32).

### 2.3 Data collection, MRI acquisition and 3D visualization

**Figure 1:**
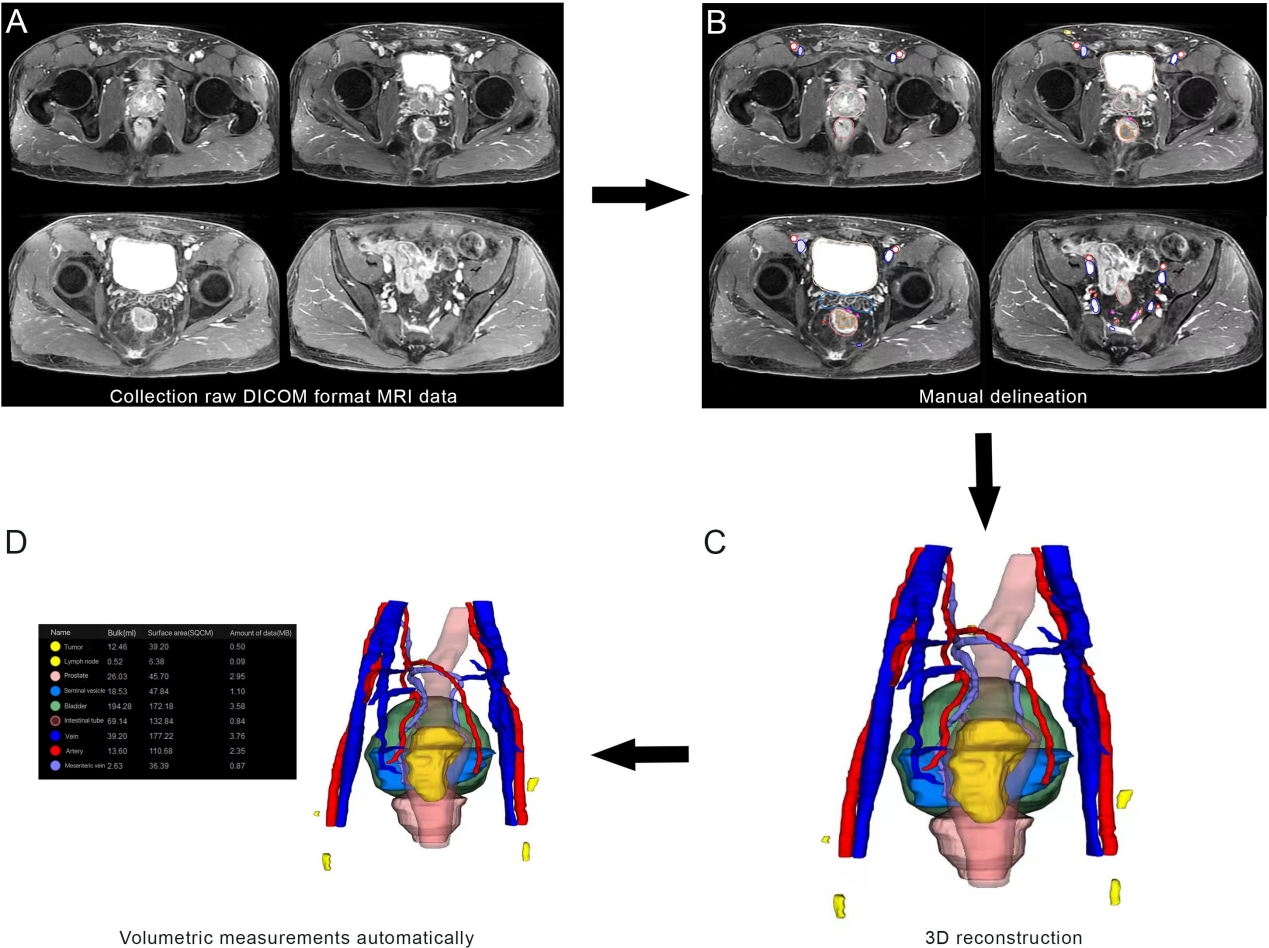
Flow diagram from magnetic resonance imaging (MRI) to three-dimensional (3D) visualization: **(A)** collection of raw DICOM format MRI data; **(B)** manual delineation; **(C)** 3D visualization; **(D)** Volumetric measurements automatically.

**Figure 2:**
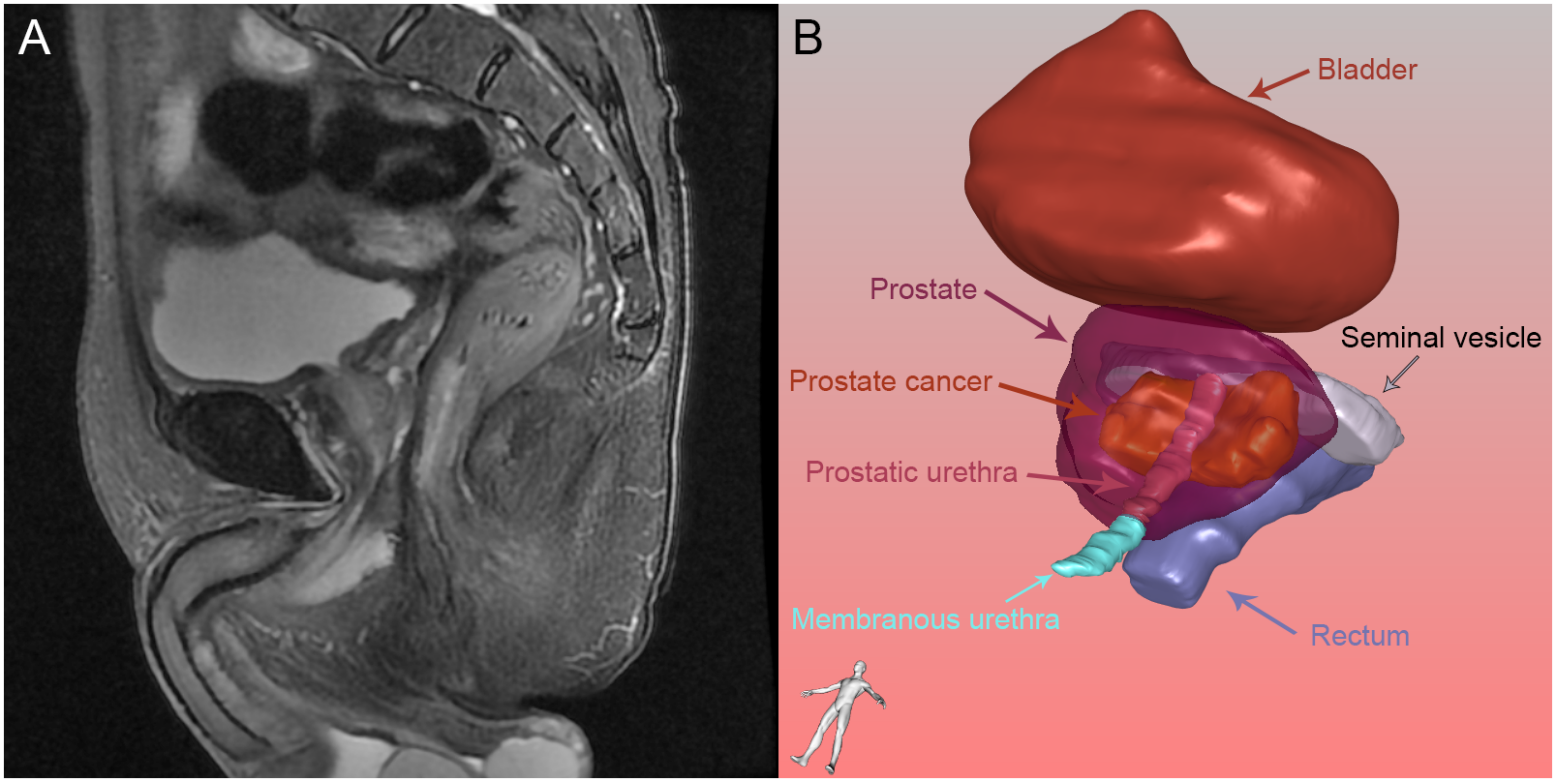
Prostate and its surrounding tissue: **(A)** Prostate and its surrounding tissue in magnetic resonance imaging; **(B)** Prostate and its surrounding tissue reconstructed by three-dimensional visualization.

Clinical and histopathological characteristics, including age, surgical approach and PSA level were collected. In accordance with the European Society of Urogenital Radiology (ESUR) guidelines and the national recommendations, mpMRI of the prostate was taken on a 3T MRI scanner with 18 or 60 channel phased-array surface-coils plus/minus 32 channel spine coil. The standardized process of MRI included T1-weighted images, T2-weighted images, diffusion-weighted images, and dynamic contrast-enhanced images. The slice thickness of MRI was 3.0-mm. Two blinded radiologists independently reviewed the MRI. The collected MRI data of patients were imported into IPS (YORKTAL) in a DICOM format file for 3D visualization of the prostate, urethra, bladder, seminal vesicle and rectum based on axial, sagittal and coronal views on MRI. Manual delineation of target organs of the urinary system was performed using IPS (YORKTAL) software. To achieve accurate visualization of the lower urinary tract anatomy, we applied corresponding 3D model-based registration to create a fusion 3D model of each axial, sagittal and coronal plane. Then, automatic and accurate measurements of target values were achieved in the 3D model. The flow diagram of 3D visualization and a drawing of the 3D visualization are shown in **Figures 1** and **2**.

### 2.4 Image analysis

**Figure 3:**
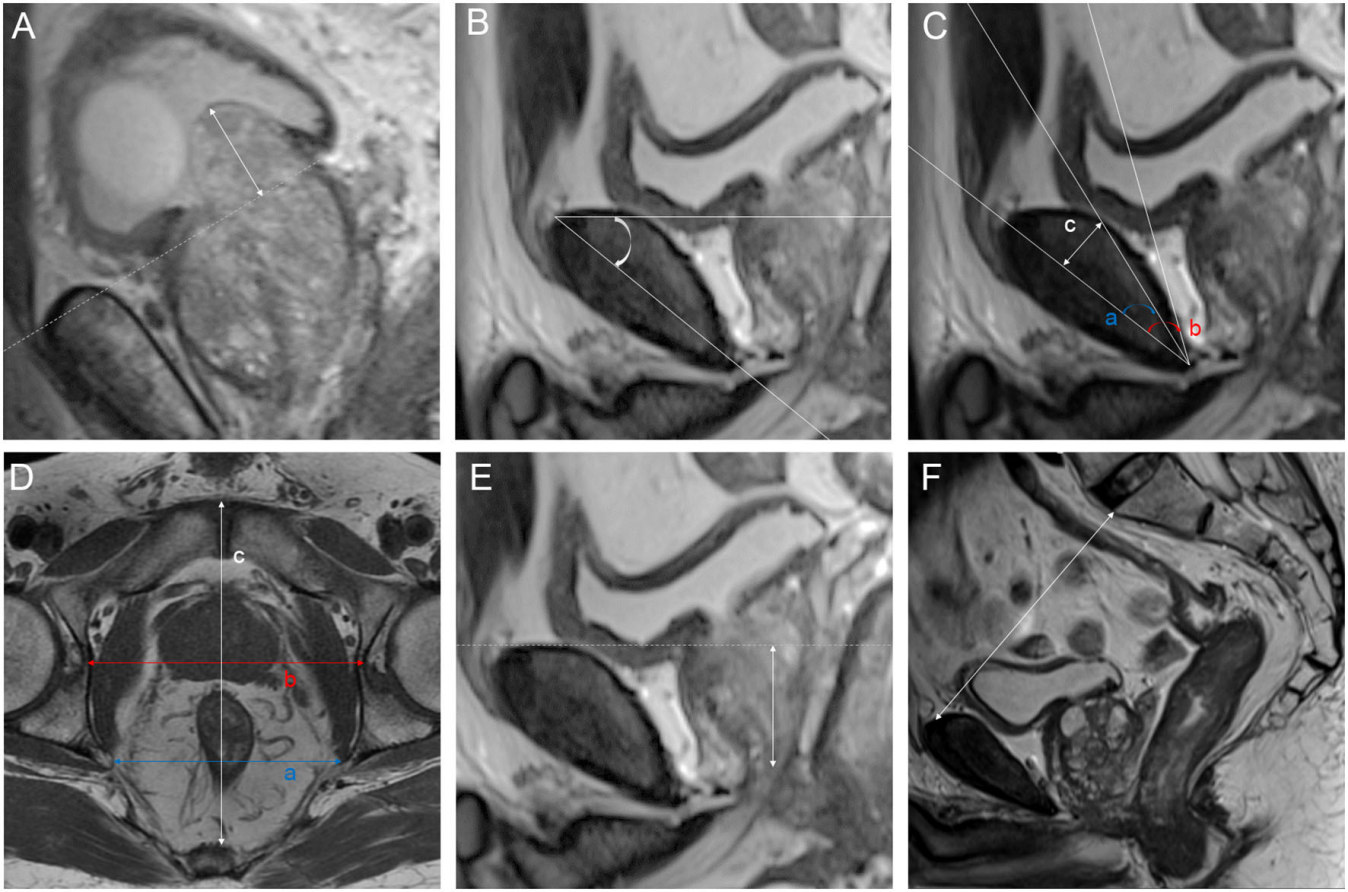
Parameters measured on magnetic resonance imaging: **(A)** bladder protrusion: the distance from the most prominent point of the prostate in the bladder to the outer boundary of the bladder wall; **(B)** symphysis angle: the angle between the long axis of the symphysis pubis and the horizontal line on the mid-sagittal image; **(C)** a: pubic angle 1: the angle between the line between the most inferior point of the pubic bone to the most bulging point of the posterior cortex and the pubic axis; b: pubic angle 2: the angle between the most inferior curve of the pubic bone and pubic axis; c: curve distance: the vertical distance from the most bulging point of the posterior cortex of the pubic bone to pubic axis; **(D)** a: interspinous distance (ISD): the distance between the bilateral ischial spines; b: bony width of the pelvis at the mid-femoral head level (BFW); c: work space AP: the distance from the anterior peritoneum to the anterior border of the coccyx (inner border); **(E)** apical depth (AD): the vertical distance from the distal margin of the prostatic apex to the horizontal line of the most proximal edge of the pubic symphysis on the midsagittal image; **(F)** pelvic brim distance: the distance from the median point of the upper margin of the pubic symphysis to the median point of the upper margin of the sacral promontory on the midsagittal image.

**Figure 4:**
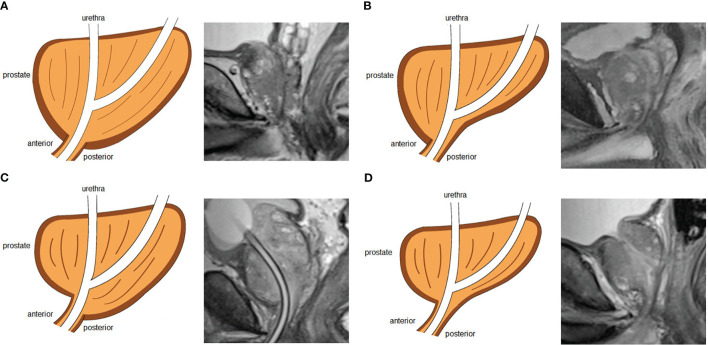
Four types of prostate apical shapes on mid-sagittal magnetic resonance imaging scan: **(A)** apical type 1: apex covering both the anterior and posterior sides of the membranous urethra; **(B)** apical type 2: apex only covering the anterior aspect of the membranous urethra; **(C)** apical type 3: apex only covering the posterior side of the membranous urethra; **(D)** apical type 4: apex not covering the membranous urethra.

**Figure 5:**
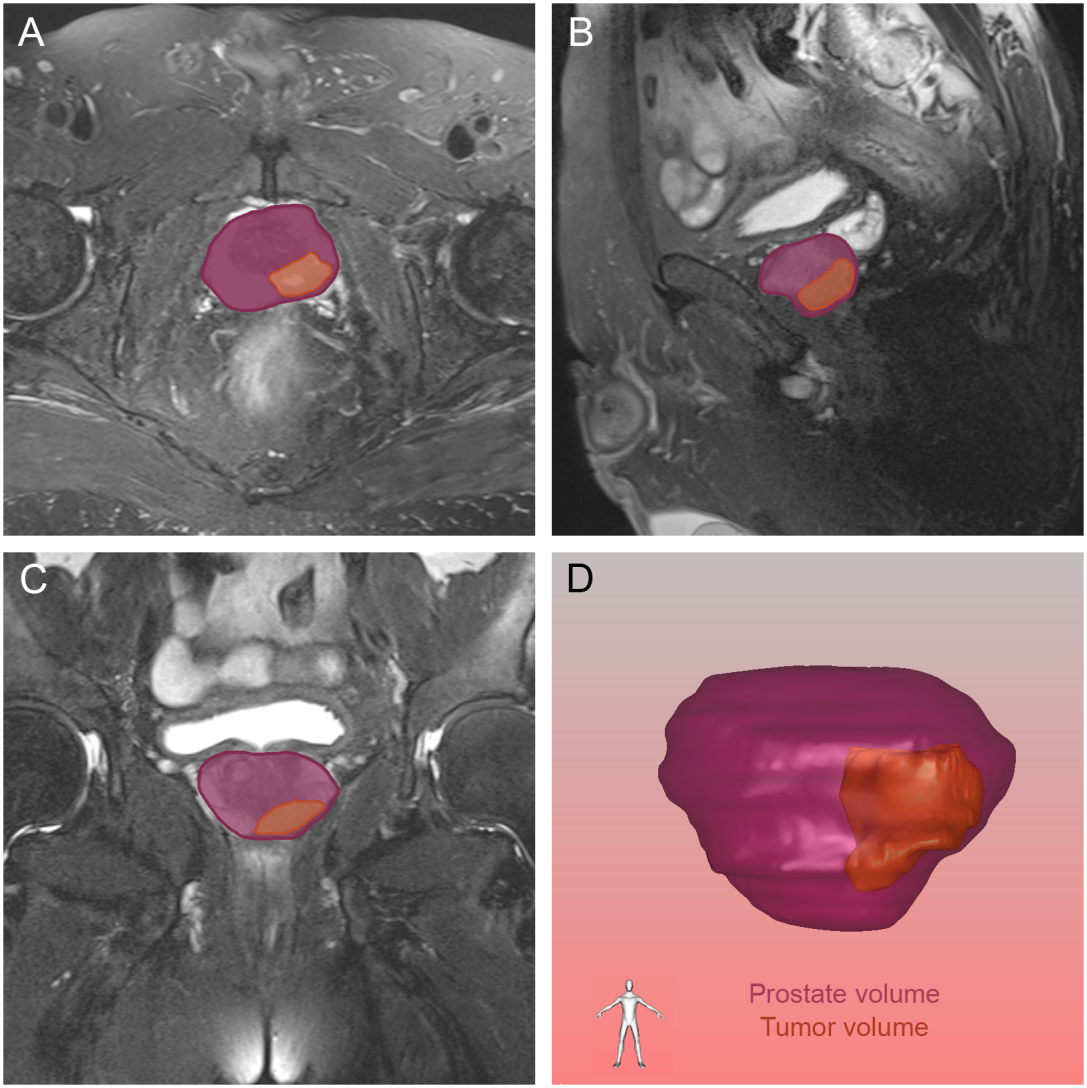
The tumor and prostate region in the axial **(A)**, sagittal **(B)** and coronal **(C)** views on magnetic resonance imaging, and prostate volume and tumor volume reconstructed by three-dimensional visualization **(D)**. The orange and purple sections indicated the prostate cancer and prostate respectively.

**Figure 6:**
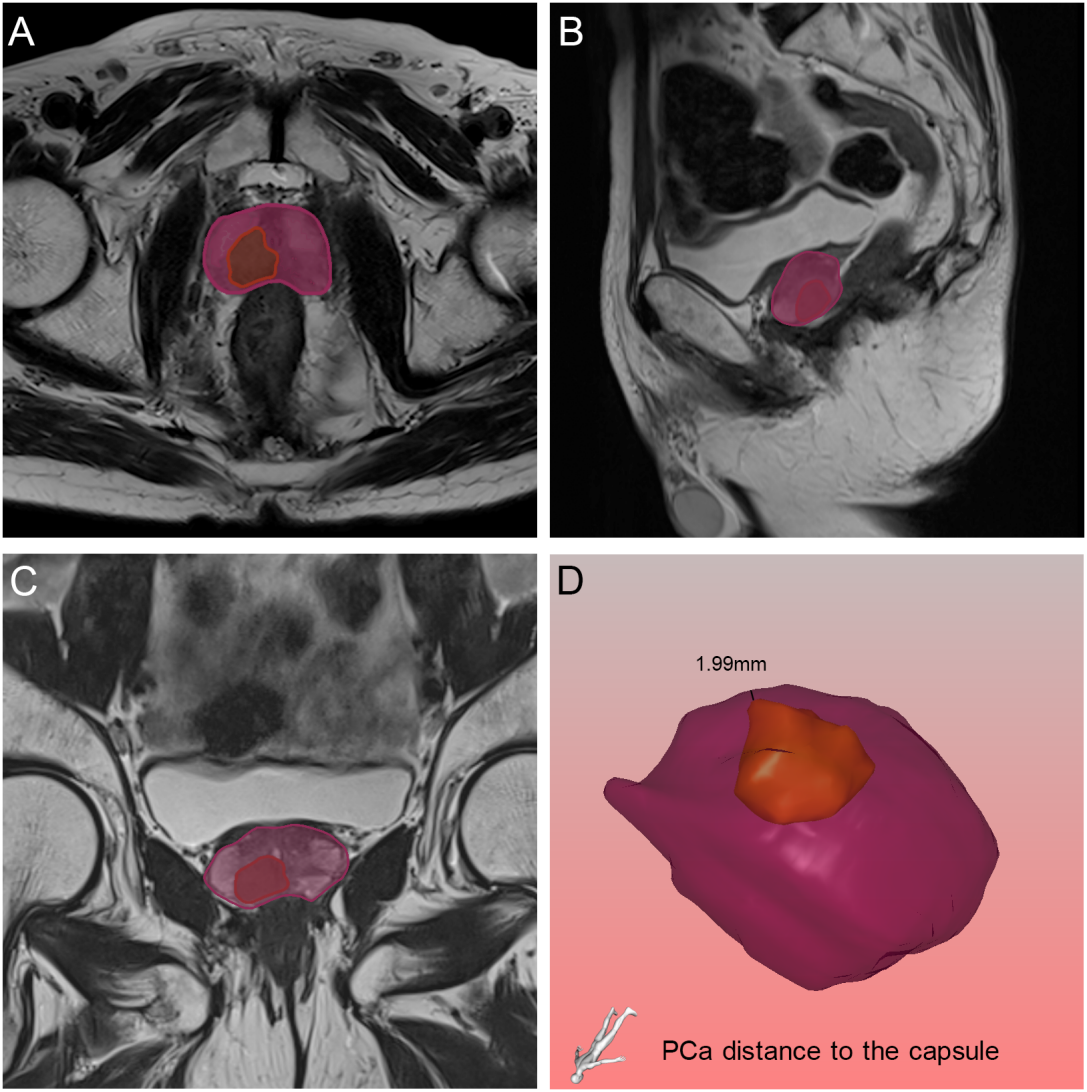
Relative position of the tumor to the capsule in axial **(A)**, sagittal **(B)** and coronal **(C)** views on magnetic resonance imaging, and the black line demonstrated the PCa distance to the capsule found by the three-dimensional visualization technique **(D)**. The orange and purple sections indicated the prostate cancer and prostate respectively.

**Figure 7:**
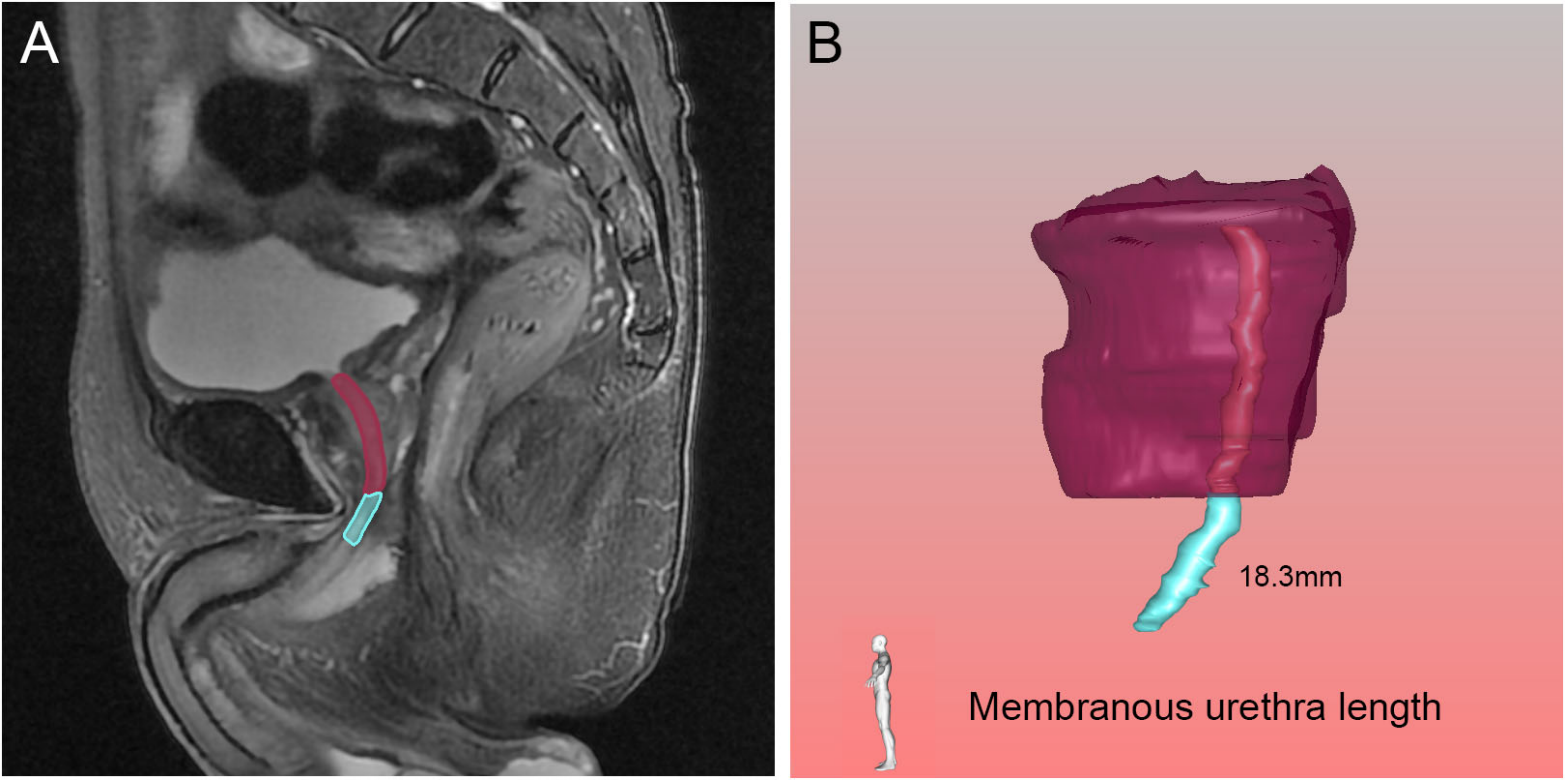
Membranous urethra length (MUL) in the sagittal **(A)** view on magnetic resonance imaging, and three-dimensional visualization technique **(B)**. The pink, blue and purple structures represented the prostatic urethra, membranous urethra and prostate respectively.

**Figure 8:**
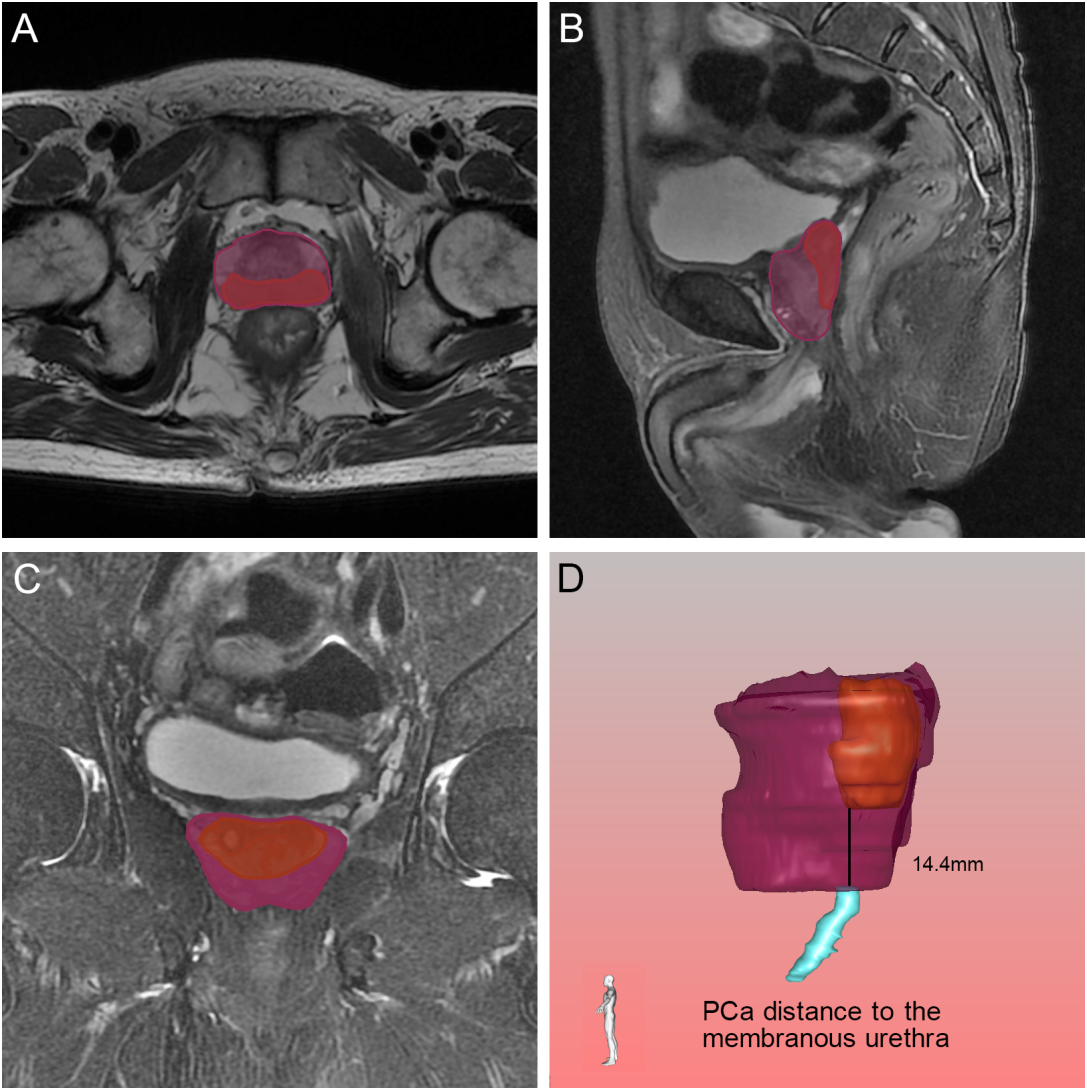
Relative position of the tumor to the membranous urethra in axial **(A)**, sagittal **(B)** and coronal **(C)** views on magnetic resonance imaging, and the black line demonstrated the PCa distance to the membranous urethra found by the three-dimensional visualization technique **(D)**. The orange, blue and purple sections indicated the prostate cancer, membranous urethra and prostate respectively.

We analyzed a total of 21 parameters based on MRI and 3D visualization. We measured 11 diameters based on osseous structures to describe the pelvic size: interspinous distance (ISD), apical depth (AD), pelvic dimension index (PDI), symphysis angle, bony width of the pelvis at the mid-femoral head level (BFW), bony width index (BWI), curve distance, pubic angle 1, pubic angle 2, the distance from the anterior peritoneum to the anterior border of the coccyx (work space AP) and pelvic brim distance. The definitions of and methods of measuring the pelvic diameters were formulated by Hong SK et al. (33). ISD was the distance between the bilateral ischial spines. The AD of the prostate was defined as the vertical distance from the distal margin of the prostatic apex to the horizontal line of the most proximal edge of the pubic symphysis on the midsagittal image. The ISD/AD ratio was defined as PDI. The symphysis angle was measured as the angle between the long axis of the symphysis pubis and the horizontal line on the midsagittal image. BFW/AD was defined as the BWI (34). Curve distance was the vertical distance from the most bulging point of the posterior cortex of the pubic bone to the pubic axis. The line between the most superior and inferior points of the pubic bone on the midsagittal image was defined as the pubic axis. Pubic angle 1 was the angle between the line between the most inferior point of the pubic bone and the most bulging point of the posterior cortex and the pubic axis. Pubic angle 2 was the angle between the most inferior curve of the pubic bone and the pubic axis. The distance from the anterior peritoneum to the anterior border of the coccyx (inner border) was defined as the work space AP (35). The pelvic brim distance was defined as the distance from the median point of the upper margin of the pubic symphysis to the median point of the upper margin of the sacral promontory on the midsagittal image (33). The measurements made on osseous structures are depicted in detail in **Figure 3**. We used the following 10 indicators to describe nonosseous structures: tumor extend (T stage), lymph node status, prostate apical shape, prostate volume (PV), tumor volume (TV), PSA density (PSAD), membranous urethra length (MUL), PCa distance to the capsule, PCa distance to the membranous urethra (UD) and bladder protrusion. We determined the T stage and lymph node status on imaging. The prostate apical shape was divided into four types as described in research by Yu YD et al. (36, 37): apical type 1: apex covering both the anterior and posterior sides of the membranous urethra; apical type 2: apex only covering the anterior aspect of the membranous urethra; apical type 3: apex only covering the posterior side of the membranous urethra; and apical type 4: apex not covering the membranous urethra (**Figure 4**). We obtained PV and TV through 3D visualization (**Figure 5**), and PSAD was also calculated based on the PV measured on 3D visualization. For PCa patients with multiple tumors, we selected the tumors closest to the location of the PSM combined with 3D visualization and pathology reports among patients with PSM. Among the patients without PSM, we selected the tumor which is closest to the capsule. We measured the distance of tumor closest to the capsule and found the PCa distance to the capsule in the 3D visualization (**Figure 6**). As the previous definition for MUL and UD, we measured these values in the 3D visualization to achieve a more accurate measurement, as presented in **Figures 7** and **8**, respectively (38). The distance between the apex of the prostate and the bulbar urethra was defined as the MUL, extending through the urogenital diaphragm. The shortest distance of the tumor to the proximal membranous urethra was defined as UD (38). Bladder protrusion was defined as the distance from the most prominent point of the prostate in the bladder to the outer boundary of the bladder wall.

### 2.5 Statistical analysis

The data obtained from MRI and 3D visualization were counted and then described by the mean and standard deviation, number of cases and percentage. SPSS 26.0 statistical software was used for statistical analysis. The t test and Mann−Whitney-Whitney test were used for continuous variables, as appropriate. Classification indicators were compared by the chi-square test or the exact probability method. Continuous variables were divided into four groups by quartile: Q1, Q2, Q3 and Q4. Then, univariate and multivariate logistic regression were performed to assess the effects of clinical variables, appraising the strength of the individual variables by calculating their ORs with their 95% confidence intervals (CIs). We conducted two-sided statistical tests and considered p values less than 0.05 as statistically significant.

We constructed the nomogram using R software version 4.3.1 with the rms package and calculated the concordance index (C-index) and the relatively corrected C-index through bootstrapping validation (1,000 bootstrap resamples). Then, we drew receiver operating characteristic (ROC) curves and calibration curves.

## 3 Results

**Figure 9:**
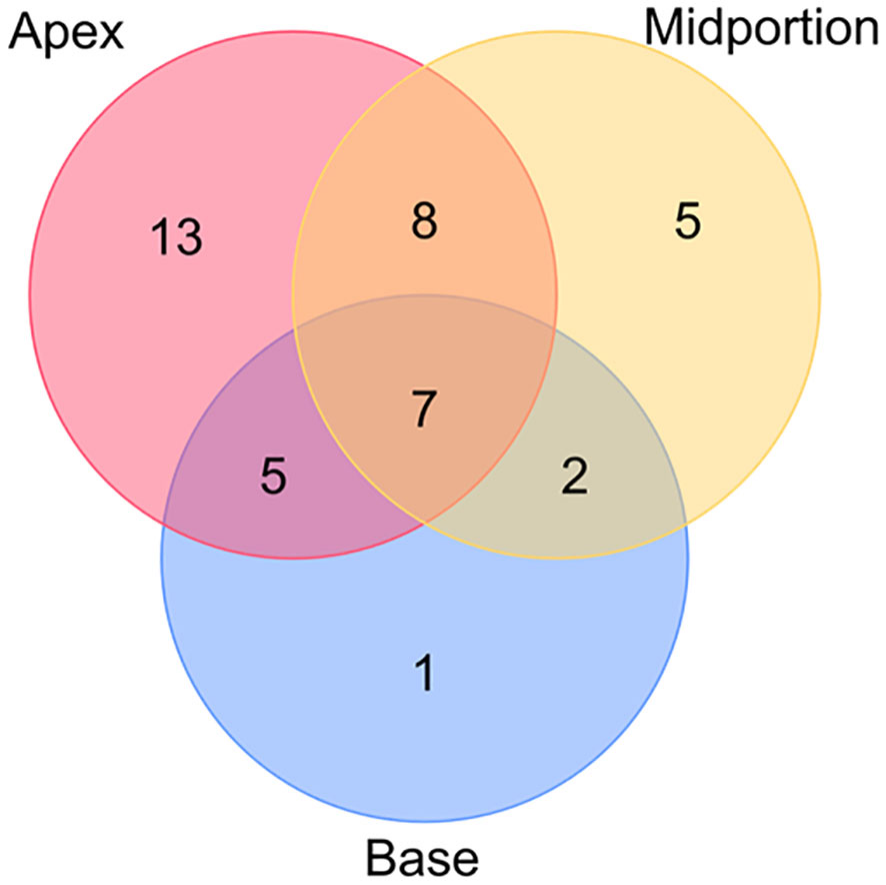
Location of PSM of 41 patients.

Among the 149 patients, 41 patients were determined to have PSM, and 22 of the 41 patients had multiple PSM sites. The PSM locations are presented in **Figure 9**. The most frequent location of PSM was the apex, accounting for 47.1% of the total PSM.

### 3.1 Clinical variables affecting PSM

#### 3.1.1 Clinical variables associated with PSM

The baseline demographic and clinical characteristics of the 41 patients with PSM and the 108 patients without PSM are shown in **Table 1**. The PSM rate was 27.5%. The results of univariate and multivariate analysis for PSM are shown in **Table 2**. We found that for the prostate apical shape, apical type 3 was significantly related to the occurrence of PSM (OR=8.262, 95% CI: 1.309-52.147, *p*=0.025), and apical type 4 was found to be a protective indicator for PSM (OR=0.070, 95% CI: 0.007-0.658, *p*=0.020). Longer UD was a protective factor of PSM, Q4 (OR=0.136, 95% CI: 0.026-0.716, *p*=0.019), referencing Q1. A larger pubic angle 2 was an independent risk factor for PSM (with Q1 as reference: Q2 OR=5.041, 95% CI: 1.095-23.203, *p*=0.038; Q3 OR=4.772, 95% CI: 1.040-21.893, *p*=0.044; Q4 OR=5.303, 95% CI: 1.818-23.807, *p*=0.029). Work space AP was identified as an independent protective factor against the occurrence of PSM, Q4 vs. Q1 having an OR=0.240 (95% CI: 0.066-0.871, *p*=0.030).

**Table 1:** Baseline demographic and clinical characteristics of the patients with positive surgical margin (PSM) and patients without PSM.

Clinical variables	Patients with PSM (n=41)	Patients without PSM (n=108)	p Value
Age (year)			
<71	15(36.6%)	56(51.9%)	0.096
≥71	26(63.4%)	52(48.1%)	
Surgical approach			
LRP	32(29.6%)	76(70.4%)	0.594
RALRP	14(34.1%)	27(65.9%)	
T stage			
≤T2	26(63.4%)	86(79.6%)	**0.041**
>T2	15(36.6%)	22(20.4%)	
Lymph node status			
N0	34(82.9%)	100(92.6%)	0.080
N1	7(17.1%)	8(7.4%)	
Prostate apical shape			
1	17(30.9%)	38(69.1%)	**0.007**
2	16(26.2%)	45(73.8%)	
3	7(58.3%)	5(41.7%)	
4	1(4.8%)	20(95.2%)	
PV (ml)			
Mean (SD)	59.60(5.05)	58.62(0.95)	0.688
Median (IQR)	52.89(36.77-68.77)	47.58(35.02-77.36)	
PSAD (ng/ml^2^)			
Mean (SD)	0.74(0.12)	0.45(0.05)	**0.004**
Median (IQR)	0.30(0.15-0.51)	0.44(0.24-0.90)	
TV (ml)			
Mean (SD)	5.35(1.16)	3.97(0.86)	**0.044**
Median (IQR)	2.01(0.66-7.72)	1.09(0.45-4.00)	
PCa distance to the capsule (mm)			
Mean (SD)	0.88(0.22)	0.80(0.13)	0.700
Median (IQR)	0.30(0.10-0.90)	0.30(0.10-0.98)	
UD (mm)			
Mean(SD)	10.54(0.92)	17.46(1.21)	**0.001**
Median (IQR)	9.80(7.11-13.65)	14.75(8.50-23.93)	
MUL (mm)			
Mean (SD)	21.61(0.88)	20.93(0.59)	0.362
Median (IQR)	21.00(17.50-25.00)	20.00(16.25-24.75)	
Bladder protrusion (mm)			
Mean (SD)	6.28(0.82)	6.80(0.65)	0.725
Median (IQR)	5.22(3.44-8.83)	5.68(3.19-8.55)	
Symphysis angle (°)			
Mean (SD)	34.83(0.73)	34.69(0.55)	0.894
Median (IQR)	34.20(31.70-38.20)	34.95(30.00-39.03)	
Curve distance (mm)			
Mean (SD)	13.65(0.40)	13.39(0.24)	0.581
Median (IQR)	14.24(11.96-15.35)	13.53(11.45-15.33)	
Pubic angle 1 (°)			
Mean (SD)	19.91(0.75)	18.71(0.41)	0.143
Median (IQR)	19.10(16.70-24.05)	18.65(15.93-21.50)	
Pubic angle 2 (°)			
Mean (SD)	30.22(1.34)	27.15(0.81)	**0.049**
Median (IQR)	29.10(23.80-34.45)	27.40(20.70-32.58)	
ISD (mm)			
Mean (SD)	102.90(1.38)	105.10(0.86)	0.182
Median (IQR)	105.00(97.50-110.50)	105.00(100.00-110.75)	
BFW (mm)			
Mean (SD)	102.73(0.87)	105.81(1.46)	0.213
Median (IQR)	102.00(100.00-106.50)	104.00(100.00-106.50)	
AD (mm)			
Mean (SD)	29.82(1.01)	30.70(0.65)	0.474
Median (IQR)	30.85(25.34-33.52)	30.85(27.25-34.74)	
PDI			
Mean (SD)	3.66(0.17)	3.62(0.10)	0.689
Median (IQR)	3.29(2.92-4.19)	3.37(3.07-4.00)	
BWI			
Mean (SD)	3.65(0.16)	3.65(0.11)	0.936
Median (IQR)	3.36(3.01-4.05)	3.38(2.94-3.95)	
Pelvic brim distance (mm)			
Mean (SD)	112.29(1.38)	113.75(0.82)	0.360
Median (IQR)	112.72(106.42-117.35)	113.38(107.60-119.64)	
Work space AP (mm)			
Mean (SD)	141.44(1.33)	144.43(1.49)	**0.032**
Median (IQR)	140.89(135.43-147.03)	144.82(139.25-150.73)	

LRP, laparoscopic radical prostatectomy; RALRP, robot-assisted laparoscopic radical prostatectomy; PV, prostate volume; PSAD, prostate-specific antigen density; TV, tumor volume; PCa, prostate cancer; UD, PCa distance to the membranous urethra; MUL, membranous urethra length; ISD, interspinous distance; BFW, bony width of the pelvis at the mid-femoral head level; AD, apical depth; PDI, pelvic dimension index; BWI, bony width index; Work space AP, anterior peritoneum to anterior border of the coccyx; SD, standard deviation; IQR, interquartile range. The bold values mean statistically significant.

**Table 2:** Univariate and multivariate analysis of clinical variables associated with positive surgical margin.

Clinical variables	Univariate		Multivariate	
	OR	p Value	OR	p Value
Age				
<71	1.0 (reference)			
≥71	1.867(0.891-3.909)	0.098		
Surgical approach				
LRP	1.0 (reference)			
RALRP	1.231 (0.572-2.650)	0.594		
T stage				
≤T2	1.0 (reference)		1.0 (reference)	
>T2	2.255 (1.024–4.966)	**0.043**	1.119(0.386-3.244)	0.836
Lymph node status				
N0	1.0 (reference)			
N1	2.574(0.868-7.627)	0.088		
Prostate apical shape				
1	1.0 (reference)	0.030	1.0 (reference)	0.006
2	0.795(0.354-1.783)	0.577	0.568(0.200-1.612)	0.288
3	3.129(0.868-11.281)	0.081	8.262(1.309-52.147)	**0.025**
4	0.112(0.014-0.902)	**0.040**	0.070(0.007-0.658)	**0.020**
PV				
Q1	1.0 (reference)	0.374		
Q2	2.057 (0.704-6.013)	0.188		
Q3	2.321 (0.801-6.728)	0.121		
Q4	1.330 (0.438-4.043)	0.615		
PSAD				
Q1	1.0 (reference)	0.020	1.0 (reference)	0.139
Q2	1.244(0.375-4.127)	0.721	0.716(0.147-3.497)	0.680
Q3	2.256(0.735-6.924)	0.155	1.079(0.229-5.087)	0.923
Q4	4.533(1.531-13.423)	**0.006**	3.757(0.737-19.157)	0.111
TV				
Q1	1.0 (reference)	0.179	1.0 (reference)	0.933
Q2	1.914(0.614-5.960)	0.263	0.830(0.180-3.815)	0.810
Q3	1.914(0.614-5.960)	0.263	0.970(0.204-4.612)	0.970
Q4	3.370(1.133-10.018)	**0.029**	0.650(0.121-3.504)	0.617
PCa distance to the capsule				
Q1	1.0 (reference)	0.630		
Q2	0.615(0.191-1.988)	0.417		
Q3	1.244(0.460-3.365)	0.667		
Q4	1.143(0.411-3.175)	0.798		
UD				
Q1	1.0 (reference)	0.007	1.0 (reference)	0.041
Q2	1.252(0.494-3.172)	0.636	1.334(0.419-4.247)	0.626
Q3	0.383(0.133-1.104)	0.076	0.656(0.188-2.286)	0.508
Q4	0.193(0.056-0.662)	**0.009**	0.136(0.026-0.716)	**0.019**
MUL				
Q1	1.0 (reference)	0.802		
Q2	1.147(0.215-6.124)	0.872		
Q3	0.794(0.114-5.518)	0.816		
Q4	0.508(0.055-4.686)	0.550		
Bladder protrusion				
Q1	1.0 (reference)	0.881		
Q2	1.493(0.539-4.136)	0.440		
Q3	1.152(0.406-3.274)	0.790		
Q4	1.111(0.392-3.149)	0.843		
Symphysis angle				
Q1	1.0 (reference)	0.630		
Q2	1.964(0.699-5.520)	0.201		
Q3	1.343(0.462-3.904)	0.588		
Q4	1.295(0.446-3.755)	0.635		
Curve distance				
Q1	1.0 (reference)	0.956		
Q2	1.152(0.406-3.274)	0.790		
Q3	1.316(0.470-3.687)	0.601		
Q4	1.267(0.454-3.541)	0.651		
Pubic angle 1				
Q1	1.0 (reference)	0.180		
Q2	1.823(0.614-5.412)	0.280		
Q3	1.069(0.344-3.322)	0.908		
Q4	2.702(0.945-7.728)	0.064		
Pubic angle 2				
Q1	1.0 (reference)	0.080		0.128
Q2	5.022(1.465-17.217)	**0.010**	5.041(1.095-23.203)	**0.038**
Q3	3.630(1.033-12.757)	**0.044**	4.772(1.040-21.893)	**0.044**
Q4	3.667(1.060-12.679)	**0.040**	5.303(1.181-23.807)	**0.029**
ISD				
Q1	1.0 (reference)	0.338		
Q2	0.608(0.228-1.627)	0.322		
Q3	0.365(0.121-1.104)	0.074		
Q4	0.583(0.224-1.517)	0.269		
BFW				
Q1	1.0 (reference)	0.496		
Q2	1.510(0.538-4.236)	0.434		
Q3	0.742(0.255-2.163)	0.583		
Q4	0.799(0.280-2.277)	0.674		
AD				
Q1	1.0 (reference)	0.473		
Q2	0.431(0.149-1.249)	0.121		
Q3	0.781(0.294-2.073)	0.620		
Q4	0.659(0.245-1.772)	0.409		
PDI				
Q1	1.0 (reference)	0.559		
Q2	0.573(0.209-1.569)	0.279		
Q3	0.509(0.181-1.432)	0.201		
Q4	0.781(0.294-2.073)	0.620		
BWI				
Q1	1.0 (reference)	0.835		
Q2	1.493(0.539-4.136)	0.440		
Q3	1.000(0.346-2.892)	1.000		
Q4	1.267(0.454-3.541)	0.651		
Pelvic brim distance				
Q1	1.0 (reference)	0.591		
Q2	0.639(0.232-1.763)	0.387		
Q3	0.725(0.275-1.914)	0.517		
Q4	0.492(0.176-1.381)	0.178		
Work space AP				
Q1	1.0 (reference)	0.190	1.0 (reference)	0.188
Q2	0.471(0.174-1.278)	0.139	0.584(0.147-2.321)	0.445
Q3	0.543(0.204-1.445)	0.221	0.599(0.171-2.103)	0.424
Q4	0.331(0.116-0.947)	**0.039**	0.240(0.066-0.871)	**0.030**

LRP, laparoscopic radical prostatectomy; RALRP, robot-assisted laparoscopic radical prostatectomy; PV, prostate volume; PSAD, prostate-specific antigen density; TV, tumor volume; PCa, prostate cancer; UD, PCa distance to the membranous urethra; MUL, membranous urethra length; ISD, interspinous distance; BFW, bony width of the pelvis at the mid-femoral head level; AD, apical depth; PDI, pelvic dimension index; BWI, bony width index; Work space AP, anterior peritoneum to anterior border of the coccyx. The bold values mean statistically significant.

#### 3.1.2 Nomogram for predicting PSM

**Figure 10:**
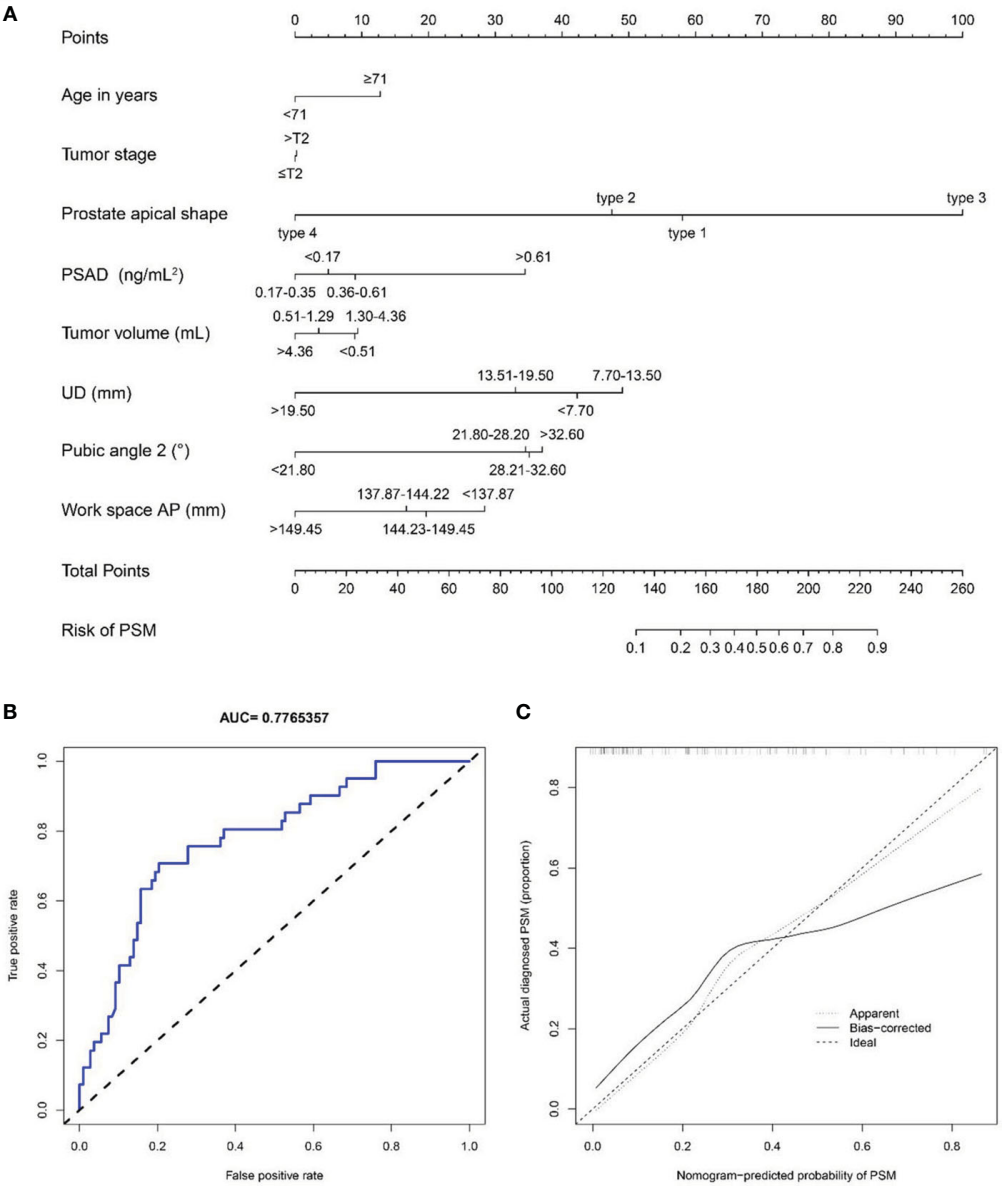
**(A)** A nomogram for predicting the probability of positive surgical margin. The predicting nomogram was based on eight different factors which established by logistic regression as well as manually selected. To use this nomogram, making a vertical line upward at each predictor to get the corresponding points, accumulating the points of all predictors and making a vertical line downward at the total points to get the probability of positive surgical margin for each patient. PSAD, prostate-specific antigen density; UD, prostate cancer distance to the membranous urethra; AP, anterior peritoneum to anterior border of the coccyx; PSM, positive surgical margin; **(B)** Receiving operating characteristic curve of the prediction nomogram of positive surgical margin; **(C)** Calibration curves for nomogram for the probability of positive surgical margin (using bootstrap resampling 1000 times).

To predict the occurrence of PSM, we constructed the nomogram shown in **Figure 10**. The area under the curve (AUC) of ROC curve and C-index of the nomogram model were equal to 0.777 and 0.845, respectively, indicating that the nomogram model made good distinctions. The corrected C-index was 0.736. The calibration curve was shown in **Figure 10**, showing the comparatively good predictive power of this machine model.

### 3.2 Clinical variables affecting APSM

#### 3.2.1 Clinical variables associated with APSM

We present the baseline demographic and clinical characteristics of the 33 patients with APSM and the 116 patients without APSM in **Table 3**. The results of univariate and multivariate logistic regression analysis are presented in **Table 4**. Longer UD was determined to be a protective factor against the occurrence of APSM, Q4 vs. Q1 having an OR=0.135 (95% CI: 0.027-0.672, *p*=0.014). Pubic angle 1 was an independent risk factor for the incidence of APSM, Q2 being correlated with a higher incidence of APSM than Q1 (OR=4.666, 95% CI: 1.049-20.749, *p*=0.043).

**Table 3:** Baseline demographic and clinical characteristics of the patients with apical positive surgical margin (APSM) and patients without APSM.

Clinical variables	Patients with APSM (n=33)	Patients with no APSM (n=116)	p Value
Age (year)			
<71	13(39.4%)	58(50.0%)	0.282
≥71	20(60.6%)	58(50.0%)	
Surgical approach			
LRP	22(66.7%)	81(69.8%)	0.729
RALRP	11(33.3%)	35(30.2%)	
T stage			
≤T2	20(60.6%)	92(79.3%)	**0.028**
>T2	13(39.4%)	24(20.7%)	
Lymph node status			
N0	28(84.8%)	106(91.4%)	0.324
N1	5(15.2%)	10(8.6%)	
Prostate apical shape			
1	14(25.5%)	41(74.5%)	0.138
2	14(23.0%)	47(77.0%)	
3	4(33.3%)	8(66.7%)	
4	1(4.8%)	20(95.2%)	
PV (ml)			
Mean (SD)	61.63(5.91)	58.11(2.80)	0.467
Median (IQR)	54.99(38.12-70.75)	47.50(35.21-75.69)	
PSAD (ng/ml^2^)			
Mean (SD)	0.76(0.14)	0.46(0.05)	**0.022**
Median (IQR)	0.44(0.24-0.98)	0.31(0.15-0.57)	
TV (ml)			
Mean (SD)	5.68(1.39)	3.97(0.81)	0.060
Median (IQR)	2.01(0.66-7.96)	1.10(0.45-4.05)	
PCa distance to the capsule (mm)			
Mean (SD)	0.72(0.19)	0.85(0.13)	0.870
Median (IQR)	0.30(0.05-0.90)	0.30(0.10-0.98)	
UD (mm)			
Mean (SD)	10.31(1.05)	17.05(1.14)	**0.002**
Median (IQR)	8.10(6.35-13.55)	14.45(8.43-23.10)	
MUL (mm)			
Mean (SD)	21.00(0.88)	21.15(0.58)	0.752
Median (IQR)	21.00(17.50-24.50)	20.00(17.00-25.75)	
Bladder protrusion (mm)			
Mean (SD)	6.53(0.98)	6.69(0.61)	0.923
Median (IQR)	5.67(3.26-9.74)	5.62(3.35-8.48)	
Symphysis angle (°)			
Mean (SD)	35.02(0.81)	34.65(0.52)	0.725
Median (IQR)	34.20(32.00-38.20)	34.95(30.03-39.03)	
Curve distance (mm)			
Mean (SD)	13.81(0.44)	13.36(0.24)	0.370
Median (IQR)	14.30(12.11-15.68)	13.53(11.45-15.16)	
Pubic angle 1 (°)			
Mean (SD)	20.46(0.83)	18.64(0.40)	**0.038**
Median (IQR)	19.50(16.90-24.30)	18.65(15.75-21.45)	
Pubic angle 2 (°)			
Mean (SD)	30.71(1.63)	27.22(0.76)	**0.038**
Median (IQR)	29.10(23.80-37.70)	27.75(21.23-32.28)	
ISD (mm)			
Mean (SD)	103.33(1.65)	104.82(0.82)	0.401
Median (IQR)	104.00(97.00-111.50)	105.00(100.00-110.00)	
BFW (mm)			
Mean (SD)	102.91(1.05)	105.54(1.37)	0.403
Median (IQR)	102.00(99.50-108.00)	103.50(100.25-107.00)	
AD (mm)			
Mean (SD)	30.65(1.12)	30.41(0.63)	0.855
Median (IQR)	31.27(27.02-35.72)	30.59(26.73-34.58)	
PDI			
Mean (SD)	3.57(0.19)	3.65(1.00)	0.240
Median (IQR)	3.23(2.84-3.69)	3.41(3.07-4.05)	
BWI			
Mean (SD)	3.55(0.18)	3.68(0.10)	0.342
Median (IQR)	3.34(2.90-3.68)	3.42(2.96-4.05)	
Pelvic brim distance (mm)			
Mean (SD)	112.07(1.64)	113.71(0.78)	0.338
Median (IQR)	112.72(106.36-117.35)	113.38(107.60-119.38)	
Work space AP (mm)			
Mean (SD)	142.11(1.51)	144.04(1.41)	0.183
Median (IQR)	143.16(136.14-147.73)	144.46(138.22-150.31)	

LRP, laparoscopic radical prostatectomy; RALRP, robot-assisted laparoscopic radical prostatectomy; PV, prostate volume; PSAD, prostate-specific antigen density; TV, tumor volume; PCa, prostate cancer; UD, PCa distance to the membranous urethra; MUL, membranous urethra length; ISD, interspinous distance; BFW, bony width of the pelvis at the mid-femoral head level; AD, apical depth; PDI, pelvic dimension index; BWI, bony width index; Work space AP, anterior peritoneum to anterior border of the coccyx; SD, standard deviation; IQR, interquartile range. The bold values mean statistically significant.

**Table 4:** Univariate and multivariate analysis of clinical variables associated with apical positive surgical margin.

Clinical variables	Univariate		Multivariate	
	OR	p Value	OR	p Value
Age				
<71	1.0 (reference)			
≥71	1.538(0.700-3.381)	0.284		
Surgical approach				
LRP	1.0 (reference)			
RALRP	1.157(0.507-2.641)	0.729		
T stage				
≤T2	1.0 (reference)		1.0 (reference)	
>T2	2.492 (1.086-5.716)	**0.031**	1.275(0.428-3.797)	0.662
Lymph node status				
N0	1.0 (reference)			
N1	1.893(0.598-5.987)	0.277		
Prostate apical shape				
1	1.0 (reference)	0.273		
2	0.872(0.372-2.043)	0.753		
3	1.464(0.382-5.619)	0.578		
4	0.146(0.018-1.193)	0.073		
PV				
Q1	1.0 (reference)	0.576		
Q2	1.425(0.441-4.608)	0.554		
Q3	2.186(0.711-6.720)	0.172		
Q4	1.378(0.427-4.446)	0.592		
PSAD				
Q1	1.0 (reference)	0.113	1.0 (reference)	0.456
Q2	1.277(0.354-4.613)	0.709	0.909(0.201-4.109)	0.901
Q3	2.121(0.637-7.069)	0.221	1.889(0.403-8.843)	0.419
Q4	3.575(1.123-11.378)	**0.031**	2.569(0.548-12.055)	0.231
TV				
Q1	1.0 (reference)	0.210	1.0 (reference)	0.853
Q2	2.652(0.737-9.546)	0.136	1.919(0.447-8.245)	0.381
Q3	2.276(0.620-8.349)	0.215	1.433(0.298-6.887)	0.653
Q4	3.808(1.099-13.195)	**0.035**	1.514(0.288-7.954)	0.624
PCa distance to the capsule				
Q1	1.0 (reference)	0.785		
Q2	0.579(0.167-2.005)	0.388		
Q3	1.042(0.365-2.972)	0.939		
Q4	0.907(0.306-2.694)	0.861		
UD				
Q1	1.0 (reference)	0.014	1.0 (reference)	0.078
Q2	0.886(0.338-2.324)	0.806	0.433(0.138-1.360)	0.152
Q3	0.288(0.090-0.919)	**0.036**	0.326(0.088-1.205)	0.093
Q4	0.158(0.041-0.616)	**0.008**	0.135(0.027-0.672)	**0.014**
MUL				
Q1	1.0 (reference)	0.935		
Q2	1.238(0.210-7.310)	0.814		
Q3	1.281(0.173-9.508)	0.809		
Q4	0.855(0.085-8.616)	0.894		
Bladder protrusion				
Q1	1.0 (reference)	0.995		
Q2	1.000(0.331-3.025)	1.000		
Q3	1.000(0.331-3.025)	1.000		
Q4	1.125(0.381-3.321)	0.831		
Symphysis angle				
Q1	1.0(reference)	0.576		
Q2	2.186(0.711-6.720)	0.172		
Q3	1.425(0.441-4.608)	0.554		
Q4	1.378(0.427-4.446)	0.592		
Curve distance				
Q1	1.0 (reference)	0.944		
Q2	1.182(0.380-3.680)	0.773		
Q3	1.378(0.452-4.196)	0.573		
Q4	1.330(0.438-4.043)	0.615		
Pubic angle 1				
Q1	1.0 (reference)	0.027	1.0 (reference)	0.044
Q2	3.077(0.864-10.954)	0.083	4.666(1.049-20.749)	**0.043**
Q3	1.176(0.290-4.774)	0.820	1.009(0.209-4.871)	0.991
Q4	4.667(1.363-15.978)	**0.014**	4.079(0.940-17.704)	0.060
Pubic angle 2				
Q1	1.0 (reference)	0.115	1.0 (reference)	0.463
Q2	3.490(0.996-12.237)	0.051	1.778(0.410-7.717)	0.442
Q3	1.650(0.424-6.418)	0.470	1.196(0.240-5.961)	0.827
Q4	3.667(1.060-12.679)	**0.040**	2.915(0.600-14.160)	0.185
ISD				
Q1	1.0 (reference)	0.305		
Q2	0.552(0.187-1.630)	0.282		
Q3	0.326(0.092-1.150)	0.081		
Q4	0.839(0.313-2.246)	0.726		
BFW				
Q1	1.0 (reference)	0.380		
Q2	1.200(0.405-3.553)	0.742		
Q3	0.429(0.125-1.469)	0.178		
Q4	0.937(0.322-2.733)	0.906		
AD				
Q1	1.0 (reference)	0.728		
Q2	0.702(0.217-2.268)	0.554		
Q3	1.343(0.462-3.904)	0.588		
Q4	1.125(0.381-3.321)	0.831		
PDI				
Q1	1.0 (reference)	0.527		
Q2	0.734(0.262-2.050)	0.555		
Q3	0.457(0.149-1.406)	0.172		
Q4	0.552(0.187-1.630)	0.282		
BWI				
Q1	1.0 (reference)	0.627		
Q2	1.534(0.535-4.398)	0.426		
Q3	0.846(0.272-2.633)	0.773		
Q4	0.819(0.263-2.543)	0.729		
Pelvic brim distance				
Q1	1.0 (reference)	0.533		
Q2	0.591(0.199-1.753)	0.343		
Q3	0.709(0.254-1.977)	0.511		
Q4	0.443(0.144-1.360)	0.155		
Work space AP				
Q1	1.0 (reference)	0.686		
Q2	0.745(0.256-2.166)	0.588		
Q3	0.868(0.305-2.466)	0.790		
Q4	0.506(0.163-1.574)	0.240		

LRP, laparoscopic radical prostatectomy; RALRP, robot-assisted laparoscopic radical prostatectomy; PV, prostate volume; PSAD, prostate-specific antigen density; TV, tumor volume; PCa, prostate cancer; UD, PCa distance to the membranous urethra; MUL, membranous urethra length; ISD, interspinous distance; BFW, bony width of the pelvis at the mid-femoral head level; AD, apical depth; PDI, pelvic dimension index; BWI, bony width index; Work space AP, anterior peritoneum to anterior border of the coccyx. The bold values mean statistically significant.

#### 3.2.2 Nomogram for predicting APSM

**Figure 11:**
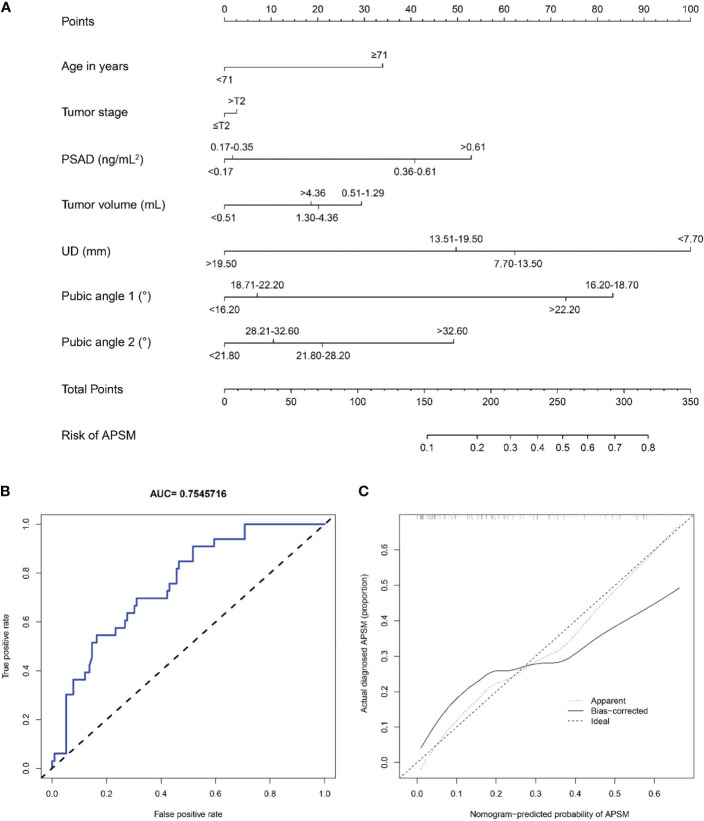
**(A)** A nomogram for predicting the probability of apical positive surgical margin. The predicting nomogram was based on seven different factors which established by logistic regression as well as manually selected. To use this nomogram, making a vertical line upward at each predictor to get the corresponding points, accumulating the points of all predictors and making a vertical line downward at the total points to get the probability of apical positive surgical margin for each patient. PSAD, prostate-specific antigen density; UD, prostate cancer distance to the membranous urethra; APSM, apical positive surgical margin; **(B)** Receiving operating characteristic curve of the prediction nomogram of apical positive surgical margin; **(C)** Calibration curves for nomogram for the probability of apical positive surgical margin (using bootstrap resampling 1000 times).

We constructed a nomogram to predict APSM, as presented in **Figure 11**. The good distinguishing ability was shown by the AUC of 0.755 and C-index of 0.799. The relatively corrected C-index was 0.676. The calibration curve of the nomogram shown in **Figure 11** presented relatively good discrimination.

## 4 Discussion

PSM is a complex issue for urologists since it is strongly associated with worse clinical outcomes (6, 39). The cutoff PSM rate in our study was 27.5%, which is high in view of the global PSM rate of 6.5-32% (6), but in the Asian region, the cutoff rate has been approximately 21.9%-38.3%, making our rate low to moderate there (19, 40–43). This phenomenon may be due to the presence of objective factors such as screening technology and the prevalence of physical examination in Asian studies, which lead to the late detection of PCa, resulting in a lower proportion of patients with an early clinical stage and thus indirectly increasing the PSM rate. Furthermore, some studies have reported that the diverse outcomes of PCa between Western and Eastern countries may be due to different pelvic anatomical structures (44–48). The most common site of PSM is the prostatic apex, with an incidence of 40-63%. Our results are in line with these, at 47.1%, due to the inclusion of multiple positive margins (19, 40, 49, 50). One reason why the apical part of the prostate is the most likely site for positive margins may be the lack of a fibrous envelope in the apical part of the prostate. The other is that freeing the distal prostate is the most challenging and thought-provoking part of the procedure in RALRP. Since the prostatic apex is located below the pubic bone, the dorsal venous complex in this area is extensive and close to the neurovascular bundle. Complete removal of the tumor by more extensive excision of the neurovascular bundle may lead to postoperative incontinence, impotence, and other lower urinary tract sequelae (51). Preservation of neurovascular bundles becomes incompatible with complete resection of the PCa. The high incidence of APSM may also be related to the surgeon’s excessive intraoperative stretching of the prostate, resulting in fissures in the prostate peritoneum and the attempt to preserve additional urethral length (52).

Since MRI is one of the most important examination methods for patients with PCa, we explored the anthropometric measurements measured on MRI. The seminal study by Permpongkosol S et al. measured the pubic angle, work space AP, work space transverse and bladder protrusion (35). These imaging parameters were analyzed, and some statistically significant anthropometric measurements were found to be associated with PSM. The reason why pubic angle 2 was strongly correlated with PSM is that the pubic angle influences the access of a laparoscopic instrument or robotic arm to the prostatic apex and thus might indirectly affect surgical outcomes. For the same reason, the longer the work space AP is, the wider the surgical space and field of view, thus lowering the overall incidence of PSM. However, it was found that ISD, BFW, pelvic brim distance, and AD in this study were not statistically significant predictors. These conclusions were probably due to the proportion of patients who underwent RALRP in our study, in whom the flexible robotic arm was able to perform more precise manipulations in a relatively narrow space than laparoscopy would have achieved.

In our study, the variation in prostate apical shape may have been strongly associated with PSM, which indicated that different prostate apical shapes would affect the difficulty of stripping the membranous urethra during the operation. Preserving the MUL as much as possible with complete tumor resection may be one of the crucial steps to avoid delayed recovery of urinary control, especially for tumors located in the apical region of the prostate. Since we uncovered a strong correlation between apical type 3 and PSM, 3D visualization for adequate evaluation was necessary before the operation, which may remind clinicians perform careful stripping of the prostatic apical stump. Owing to the fact that apical type 4 did not enclose a membranous urethra at apex, this gives the urologist a maximum likelihood of completely removing the prostate and preserving adequate MUL, which may contribute to explaining why patients with apical type 4 had a better prognosis, a finding consistent with previous studies (36). In addition, finding effective surgical techniques or methods for stripping the apex of the prostate may reduce the high incidence of PSM and APSM, such as adjusting the robotic lens to face 30° upward and pulling the prostate cephalad, dissecting from the posterior inferior surface of the gland to the junction of the acinus and the membranous urethra, and early dissection of the dorsal venous complex without ligation by adding pneumoperitoneum (53, 54).

Although many meaningful parameters were identified in the MRI in this study, due to the existence of objective factors such as the limitation of viewing angle in conventional MRI, our study innovatively focused on 3D visualization techniques to more finely measure the diameters and volumes that cannot be measured or measured accurately in 2D images. For the accurate measurement of prostate volume, for instance, most medical institutions today use the elliptical formula for estimation to substitute actual data. However, this method is fundamentally flawed because the formula only obtains the appropriate data for estimation from three dimensions (sagittal, coronal and axial images). Additionally, due to the presence of tumors in PCa patients, the irregular shape of the prostate caused by the interaction of various stresses in the prostate, the diversity of tumor locations and the great individual differences in the prostate, the deviation in measurements of the prostate in patients with PCa using the conventional method will be even greater, reducing the clinical relevance. It should be noted that the interpretation of this result is somewhat limited by the fact that some patients contained multiple prostate tumors, and there were particular restrictions on tumor selection. The above results are consistent with the findings of Song C et al., who also mentioned limitations in tumor volume measurements and utilized calculated volumes rather than actual volumes. Our study has overcome the above limitation with 3D technology, making the results more convincing (55). The reason that no significance was found in the multivariate analysis might be due to the larger tumor having closer proximity to the prostate apex, which is another important finding of ours: patients with a longer UD had a lower incidence of PSM and APSM. Quentin M et al. similarly also found the above result (38). Considering the recovery of early urinary control in patients, the surgeon will try to preserve the urethral length when releasing the prostate apex, which may indirectly contribute to the higher likelihood of PSM and APSM in patients with a shorter UD. We recommend preoperative 3D visualization for patients with a potentially short UD identified by MRI, which, by looking at the relative position of the tumor to the membranous urethra from more planes, enables a highly individualized preoperative surgical plan and reduces the likelihood of PSM by widening the extent of the membranous urethra to be resected.

Only a few previous studies have performed nomograms for the preoperative prediction of PSM, and only correlations regarding clinical information and conventional MRI have been explored (14, 56). In this study, 3D visualization technology was combined with significant clinical data and imaging parameters based on MRI to improve the predictive capability and accuracy of the nomograms. The AUC and C-index values both demonstrated a great capability of prediction. Therefore, urologists can predict the likelihood a patient will suffer from PSM and APSM preoperatively, thus reducing the occurrence of events during surgery by applying neoadjuvant therapy or expanding the scope of surgical resection.

This study had a few limitations that should be considered. First, it was conducted in a single center, resulting in a limited sample size as well as a lack of external validation. Second, due to the most of the included patients lived in northeastern China, selection bias and potential confounding factors may have influenced our conclusions. Furthermore, we conducted the retrospective clinical study from December 2016 to April 2022. During this time period, all the patients in our center were scanned with a 3.0-mm-thick layer, which may affect the quality of the 3D visualization. As the layer thickness of the scans may affect the observation of detailed anatomy, thin-slice MRI would improve the quality of 3D visualization more accurate. In order to verify the conclusions of this study and the power of the independent risk factors and nomograms for predicting PSM and APSM, large-scale, multicenter, prospective studies are warranted. Moreover, an external validation cohort is needed to verify our findings. By utilizing 3D visualization techniques based on thin-slice imaging, more potentially instructive factors can be measured in more accurate images, providing clinical guidance for the subsequent development of imaging guidelines for prostate PSM prediction. 3D visualization can help observe tumor and surrounding environment changes more intuitively and assess treatment efficacy more effectively after endocrine therapy or chemotherapy. We further make adjustment for therapeutic regimen timely based on the precise monitoring the changes of the degree of tumor volumetric or the size of pelvic lymph nodes. Although our study did not conclude that MUL can act as an independent influencing factor for PSM, we are the first to study MUL using 3D visualization. In other aspects of digital medicine, we have embarked on deep learning of 2D and 3D imaging images using artificial intelligence with the aim of quickly predicting the likelihood of PSM preoperatively. Through more in-depth research on this topic, we will explore ways to control PSM as well as APSM from various dimensions to help reduce PSM and APSM rates, improve patient prognoses, and enhance the quality of life of PCa survivors in China and around the world.

## 5 Conclusion

In conclusion, our findings show that UD, work space AP, prostate apical shape and public angle 2 are significant predictors of PSM and that UD and pubic angle 1 are independent factors for APSM. The constructed nomograms performed well at predicting these events. The routine use of 3D visualization in clinical diagnosis can help to tailor individual treatment regimens to benefit PCa patients. We will validate the current findings and reveal the mystery in future prospective large-sample multicenter studies by utilizing higher-quality 3D technology and investigating the risk factors for PSM and APSM in greater depth to provide clinical guidance for the development of imaging guidelines for PSM and APSM prediction.

## Data availability statement

The original contributions presented in the study are included in the article/supplementary material. Further inquiries can be directed to the corresponding authors.

## Ethics statement

The studies involving humans were approved by the Ethics review committee of the Second Affiliated Hospital of Dalian Medical University (ethical approval number: 202285). The patients/participants provided their written informed consent to participate in the study. Written informed consent was obtained from the individual(s) for the publication of any potentially identifiable images or data included in this article.

## Author contributions

BF, ZL, LW and JL contributed to the study concept and design, undertook project leadership and guaranteed this work. LZ, YCW and were responsible for the acquisition of data. YTW, HW, ZX and XD analyzed data and interpreted the data. LZ and YCW controlled quality of data and algorithms. BF, LZ and YCW wrote the first draft of the manuscript. BF, ZL, LW and ZD reviewed and revised the manuscript. HP and JX provided technical support. All authors contributed to the article and approved the submitted version.
